# The biochemistry of belief

**DOI:** 10.4103/0019-5545.58285

**Published:** 2009

**Authors:** T.S. Sathyanarayana Rao, M. R. Asha, K. S. Jagannatha Rao, P. Vasudevaraju

**Affiliations:** Department of Psychiatry, JSS University, JSS Medical College Hospital, M.G. Road, Mysore - 570 020, India; 1 Reiki Practitioner and Freelance Writer, 788/160, 18^th^ Cross, Ramanuja Road, Mysore - 570 020, India; 2 Departments of Biochemistry and Nutrition, Central Food Technological Research Institute, Mysore - 570 020, India

***“Man is what he believes”***

***Anton Chekhov***

Beliefs are basically the guiding principles in life that provide direction and meaning in life. Beliefs are the preset, organized filters to our perceptions of the world (external and internal). Beliefs are like ‘Internal commands’ to the brain as to how to represent what is happening, when we congruently believe something to be true. In the absence of beliefs or inability to tap into them, people feel disempowered.

Beliefs originate from what we hear - and keep on hearing from others, ever since we were children (and even before that!). The sources of beliefs include environment, events, knowledge, past experiences, visualization etc. One of the biggest misconceptions people often harbor is that belief is a static, intellectual concept. Nothing can be farther from truth! Beliefs are a choice. We have the power to choose our beliefs. Our beliefs become our reality.

Beliefs are not just cold mental premises, but are ‘hot stuff’ intertwined with emotions (conscious or unconscious). Perhaps, that is why we feel threatened or react with sometimes uncalled for aggression, when we believe our beliefs are being challenged! Research findings have repeatedly pointed out that the emotional brain is no longer confined to the classical locales of the hippocampus, amygdala and hypothalamus.[1] The sensory inputs we receive from the environment undergo a filtering process as they travel across one or more synapses, ultimately reaching the area of higher processing, like the frontal lobes. There, the sensory information enters our conscious awareness. What portion of this sensory information enters is determined by our beliefs. Fortunately for us, receptors on the cell membranes are flexible, which can alter in sensitivity and conformation. In other words, even when we feel stuck ‘emotionally’, there is always a biochemical potential for change and possible growth. When we choose to change our thoughts (bursts of neurochemicals!), we become open and receptive to other pieces of sensory information hitherto blocked by our beliefs! When we change our thinking, we change our beliefs. When we change our beliefs, we change our behavior.

A mention of the ‘Placebo’ is most appropriate at this juncture. Medical history is replete with numerous reported cases where placebos were found to have a profound effect on a variety of disorders. One such astounding case was that of a woman suffering from severe nausea and vomiting. Objective measurements of her gastric contractions indicated a disrupted pattern matching the condition she complained of. Then a ‘new, magical, extremely potent’ drug was offered to her, which would, the doctors proclaimed, undoubtedly cure her nausea. Within a few minutes, her nausea vanished! The very same gastric tests now revealed normal pattern, when, in actuality, she had been given syrup of ipecac, a substance usually used to *induce* nausea! When the syrup was presented to her, paired with the strong suggestion of relief of nausea, by an authority figure, it acted as a (command) message to the brain that triggered a cascade of self-regulatory biochemical responses within the body.[2] This instance dramatically demonstrates that the influence of placebo could be more potent than expected drug effect.

An important observation was that, part of the placebo response seemed to involve the meaning of the disorder or the illness to the individual. In other words, the person's belief or how she/he interprets (inter-presents or internally represents) directly governs the biological response or behavior. Another remarkable study involved a schizophrenic. This woman was observed to have split personality. Under normal conditions, her blood glucose levels were normal. However, the moment she believed she was diabetic, her entire physiology changed to become that of a diabetic, including elevated blood glucose levels.[3]

Suggestions or symbolic messages shape beliefs that in turn affect our physical well being. Several cases of ‘Disappearance of warts’ have been reported by Ornstein and Sobel wherein they ponder on how the brain translates the suggestions (sometimes using hypnosis) into systematic biochemical battle strategies such as chemical messengers sent to enlist the aid of immune cells in an assault on the microbe-induced miniature tumor or probably small arteries are selectively constricted, cutting off the vital nutrient supply to warts but not touching the neighboring healthy cells.[2]

Findings of carefully designed research indicate that our interpretation of what we are seeing (experiencing) can literally alter our physiology. In fact, all symptoms of medicine work through our beliefs. By subtly transforming the unknown (disease/disorder) into something known, named, tamed and explained, alarm reactions in the brain can be calmed down. All therapies have a hidden, symbolic value and influence on the psyche, besides the direct specific effect they may have on the body.

Just as amazingly life-affirming placebos are, the reverse, “Nocebo' has been observed to be playing its part too. It is associated with negative, life-threatening or disempowering beliefs. Arthur Barsky, a psychiatrist states that it is the patient's expectations – beliefs whether a drug or procedure works or will have side effects – that plays a crucial role in the outcome.[4]

The biochemistry of our body stems from our awareness.[5] Belief-reinforced awareness becomes our biochemistry. Each and every tiny cell in our body is perfectly and absolutely *aware* of our thoughts, feelings and of course, our beliefs. There is a beautiful saying ‘Nobody grows old. When people stop growing, they become old’. If you believe you are fragile, the biochemistry of your body unquestionably obeys and manifests it. If you believe you are tough (irrespective of your weight and bone density!), your body undeniably mirrors it. When you believe you are depressed (more precisely, when you become consciously aware of your ‘Being depressed’), you stamp the raw data received through your sense organs, with a judgment – that is your personal view – and physically become the ‘interpretation’ as you internalize it. A classic example is ‘Psychosocial dwarfism’, wherein children who feel and *believe* that they are unloved, translate the perceived lack of love into depleted levels of growth hormone, in contrast to the strongly held view that growth hormone is released according to a preprogrammed schedule coded into the individual's genes!

Providing scientific evidence to support a holistic approach to well being and healthcare, Bruce Lipton sheds light on mechanism underlying healing at cellular level. He emphasizes that ‘love’ is the most healing emotion and ‘placebo’ effect accounts for a substantial percentage of *any* drug's action, underscoring the significance of beliefs in health and sickness. According to him, as adults, we still believe in and act our lives out based on information we absorbed as children (pathetic indeed!). And the good news is, we can do something about the ‘tape’ our subconscious mind is playing (ol' silly beliefs) and change them NOW.[6] Further recent literature evidences provided knowledge based on scientific principles of biology of belief. There are limited studies on clinics of traditional beliefs and if we get more scientific data, we can use these traditional systems in clinical mental health management. Human belief system is formed by all the experiences learned and experimented filtered through personality.[7] The senses to capture inner and outer perceptions have higher brain potentials. Some questions that arise in this context are, does the integration and acceptance of these perceptions result in the establishment of beliefs? Does the establishment of these beliefs depend on proof demonstrations? The proofs might be the perceptions, which we can directly see or having scientific proof or custom or faith.[8,9] Beliefs are developed as stimuli received as trusted information and stored in the memory. These perceptions are generalized and established into belief. These beliefs are involved in the moral judgment of the person. Beliefs help in decision-making. Bogousslavsky and Inglin explained that, how some physicians were more successful by taking an account of patient beliefs. Beliefs influence factors involved in the development of psychopathology. They also influence the cognitive and emotional assessment, addictiveness, responses to false positives and persistent normal defensive reactions. Total brain function is required in stabilizing the belief and in responding to environmental system. Some of the brain regions and the neural circuits are very important in establishing beliefs and executing emotions. Frontal lobes play a major role in beliefs. Mental representations of the world are integrated with sub-cortical information by prefrontal cortex. Amygdala and Hippocampus are involved in the process of thinking and thus help in execution of beliefs. NMDA receptor is involved in thinking and in the development of beliefs. These beliefs are subjected to challenge. A belief that is subjected to more challenges becomes stronger. When a new stimulus comes, it creates distress in the brain with already existing patterns. The distress results in the release of dopamine (neurotransmitter) to transmit the signal.[10,11] Research findings of Young and Saxe (2008) revealed that medial prefrontal cortex is involved in processing the belief valence.[12] Right temporoparietal junction and precuneus are involved in the processing of beliefs to moral judgment. True beliefs are processed through right temporoparietal junction.[13,14] Saxe (2006) explained that beliefs judging starts at the age of five years citing example of judging of belief questions on short stories by the children.[15] Belief attribution involved activating regions of medial prefrontal cortex, superior temporal gyri and hippocampal regions. Studies by Krummenacher *et al*, have shown that dopamine levels are associated with paranormal thoughts suggesting the role of dopamine in belief development in the brain.[16] Flannelly *et al*, illustrated on how primitive brain mechanisms that evolved to assess environmental threats in related psychiatric disorders.[17] Also were highlighted the issues such as the way beliefs can affect psychiatric symptoms through these brain systems. The theories discussed widely are related to (a) link psychiatric disorders to threat assessment and (b) explain how the normal functioning of threat assessment systems can become pathological. It is proposed that three brain structures are implicated in brain disorders in response to threat assessment and self-defense: the regions are the prefrontal cortex, the basal ganglia and parts of limbic system. The functionality of these regions has great potential to understand mechanism of belief formation and its relevance in neurological functions/dysfunctions. Now it is clear that biology and physiology of belief is an open area for research both at basic and clinical level. The future directions are to develop validated experimental or sound theoretical interpretation to make ‘BELIEF’ as a potential clinical management tool.

Perceptual shifts are the prerequisites for changing the belief and hence changing the biochemistry of our body favorably. Our innate desire and willingness to learn and grow lead to newer perceptions. When we consciously allow newer perceptions to enter the brain by seeking new experiences, learning new skills and changed perspectives, our body can respond in newer ways –this is the true secret of youth. Beliefs (internal representations/interpretations) thus hold the magic wand of remarkable transformations in our biochemical profile. If you are chasing joy and peace all the time everywhere but exclaim exhausted, ‘Oh, it's to be found nowhere!’, why not change your interpretation of NOWHERE to ‘NOW HERE’; just by introducing a gap, you change your awareness – that changes your belief and that changes your biochemistry in an instant!

Everything exists as a ‘Matrix of pure possibilities’ akin to ‘formless’ molten wax or moldable soft clay. We shape them into anything we desire by choosing to do so, prompted, dictated (consciously or *un*consciously) by our beliefs. The awareness that we are part of these ever-changing fields of energy that constantly interact with one another is what gives us the key hitherto elusive, to unlock the immense power within us. And it is our awareness of this awesome truth that changes everything. Then we transform ourselves from passive onlookers to powerful creators. Our beliefs provide the script to write or *re*-write the code of our reality.

Thoughts and beliefs are an integral part of the brain's operations. Neurotransmitters could be termed the ‘words’ brain uses to communicate with exchange of information occurring constantly, mediated by these molecular messengers. Unraveling the mystery of this molecular music induced by the magic of beliefs, dramatically influencing the biochemistry of brain could be an exciting adventure and a worth pursuing cerebral challenge.
